# Market shocks, climate vulnerability, and income loss in informal indigenous food systems: evidence from street vendors in Durban, South Africa

**DOI:** 10.3389/fnut.2025.1621204

**Published:** 2025-09-03

**Authors:** Xolile Mkhize, Thobani Cele

**Affiliations:** Department of Community Extension, Faculty of Applied and Health Sciences, Mangosuthu University of Technology, Durban, South Africa

**Keywords:** indigenous food crops, street vendor resilience, informal food markets, climate change resilience (SDG 13), food security (SDG 2), eThekwini municipality-Durban

## Abstract

Indigenous food crops are vital for nutrition security, climate resilience, and inclusive livelihoods, aligning with Sustainable Development Goals 2 (Zero Hunger), 8 (Decent Work and Economic Growth), and 13 (Climate Action). Yet, the street vendors who distribute these crops remain highly vulnerable to overlapping socio-environmental disruptions. This study investigates how compound shocks (the 2022 Durban floods, the COVID-19 pandemic, and the 2021 civil unrest) affect the economic sustainability of indigenous food crop vendors in South Africa. A cross-sectional survey of 34 vendors at Durban’s Warwick Early Morning Market was conducted, with data analyzed using descriptive statistics, and robust regression modeling. Results show that 88% of vendors experienced income loss due to flooding, 71% due to COVID-19, and 68% due to unrest. The regression model identified lack of cold storage (*β* = 1.53, *p* < 0.01), crop perishability (*β* = 1.10, *p* < 0.05), and exposure to these shocks as statistically significant predictors of income reduction. These findings highlight the urgent need for inclusive urban food governance, investment in post-harvest infrastructure, and support for informal vendors as key actors in achieving SDG-aligned, climate-resilient food systems.

## 1 Introduction

The sustainable preservation of indigenous food crop markets is essential for achieving several global development goals, particularly Sustainable Development Goal (SDG) 2: Zero Hunger, SDG 8: Decent Work and Economic Growth, and SDG 13: Climate Action. These markets contribute significantly to enhancing food and nutrition security, preserving cultural heritage, and promoting economic resilience, especially in low-income urban communities across the Global South (1, 2). In many African cities, informal food systems anchored by street vendors serve as the primary source of affordable, fresh produce for poor households (3). Street vendors operating from spaces such as the Warwick Early Morning Market (linked to the Durban Fresh Produce Market) serve as vital nodes in the city’s food distribution network (4). Despite limited infrastructure, these markets redistribute produce in quantities aligned with household needs, using adjusted packaging and pricing. They also act as cultural hubs, preserving knowledge on indigenous foods and maintaining social traditions of food preparation and consumption (4).

In South Africa, and particularly within the eThekwini Metropolitan Municipality in Durban, street vendors are indispensable to the informal food economy (4). They act as intermediaries between rural producers and urban consumers, facilitating access to under-commercialized, nutrient-dense indigenous food crops that are largely absent from formal retail chains (4). Crops such as *Ipomoea batatas* (known as *ubhatata* in isiZulu language), *Colocasia esculenta* (isiZulu language*: amadumbe*), Amaranthus spp. (isiZulu language*: imbuya*), *Cucurbita maxima* (isiZulu language*: imfino yezintanga*), Momordica foetida (isiZulu language*: intshungu*), and *Arachis hypogaea* L. (isiZulu language*: amantongomane*) form a vital part of Durban’s informal food landscape due to their rich nutritional profile, including essential micronutrients, dietary fiber, and plant-based proteins (5, 6). These indigenous crops are highly adaptable to harsh climatic conditions, making them suitable for climate-smart agriculture and long-term adaptation strategies (7, 8). Despite these benefits, their integration into mainstream food markets remains limited. Factors such as inadequate cold storage, poor post-harvest handling, limited agro-processing capacity, and weak consumer demand contribute to high spoilage rates, reduced profitability, and market invisibility (9, 10). Vendors often resort to selling perishable crops at reduced prices to avoid total losses, a situation exacerbated by a lack of training in food safety, financial literacy, and business management (11).

While significant research has been conducted on the income-generating potential of smallholder farmers under climate and market variability such as (12–14), relatively little is known about the income losses suffered by informal street vendors during such shocks. Existing studies tend to focus on productivity, yield, or gross earnings of rural producers (15), neglecting the compounded vulnerabilities experienced by urban vendors during floods, unrest, or pandemics. This study addresses a critical gap in the literature by examining how climate vulnerability, market disruptions, and infrastructural limitations affect the economic sustainability of indigenous food crop vendors in a high-risk urban environment. While much research has focused on producers and formal agricultural value chains, informal street vendors who play a pivotal role in ensuring food access for low-income urban households remain largely overlooked. This study specifically investigates how environmental and socio-economic stressors impact the income, market participation, and infrastructure access of indigenous crop vendors in Durban, South Africa. The research is theoretically grounded in discourses on the informal economy and climate adaptation, with particular emphasis on the structural exclusion of informal actors from urban food governance. The study aims to: (1) profile the socio-economic characteristics of indigenous vendors; (2) identify key market and climate shocks contributing to income loss and vulnerability; and (3) explore how storage limitations, perishability, and external shocks constrain their participation and sustainability within urban informal food systems.

## 2 Literature review

### 2.1 Theoretical and conceptual foundations of informal food system vulnerability

Urban informal food systems, particularly in African cities, operate within complex networks of economic survival, social exchange, and marginal regulatory inclusion (16–18). Theoretical framework on informality and street vending are largely rooted in broader frameworks of the informal economy, particularly in relation to the state and urban development (19). Three dominant schools of thought shape this discourse: dualism, which sees informality as a transitional stage in economic growth; legalism, which attributes informality to burdensome regulatory environments; and structuralism, which underscores the unequal and often exploitative dynamics between the formal and informal sectors (20, 21).

Theoretical literature on urban food governance increasingly recognizes street vendors as essential, yet marginalized, actors within informal food systems (22). These frameworks highlight how vendors operate in regulatory gray zones, often excluded from formal planning, infrastructure, and decision-making processes (23). Scholars argue that inclusive governance models must acknowledge the embeddedness of street vendors in urban food security and actively integrate them into policy and infrastructure development (24).

Vulnerability frameworks, particularly those informed by sustainable livelihoods and urban risk theory, conceptualize street vendors’ exposure to multiple stressors such as economic, environmental, and institutional as shaped by both shocks and structural exclusions (25, 26). For informal food vendors, vulnerability stems not only from acute events such as flooding or pandemics, but also from chronic risks like insecure tenure, exclusion from infrastructure planning, and lack of policy recognition (27). These frameworks argue that building resilience requires addressing both immediate threats and the underlying drivers of marginalization embedded in urban governance systems (4). These frameworks suggest that vulnerability is not only the result of environmental shocks but also of institutional and infrastructural disconnection. In the context of street vendors, their place in the value chain is often fragmented, with weak connections to storage, credit, and processing systems. This study draws on these theories to analyze how market constraints, climate risk, and urban governance shape the lived experiences of informal vendors.

#### 2.1.1 Conceptual framework: intersecting shocks and systemic vulnerabilities

**Figure 1:**
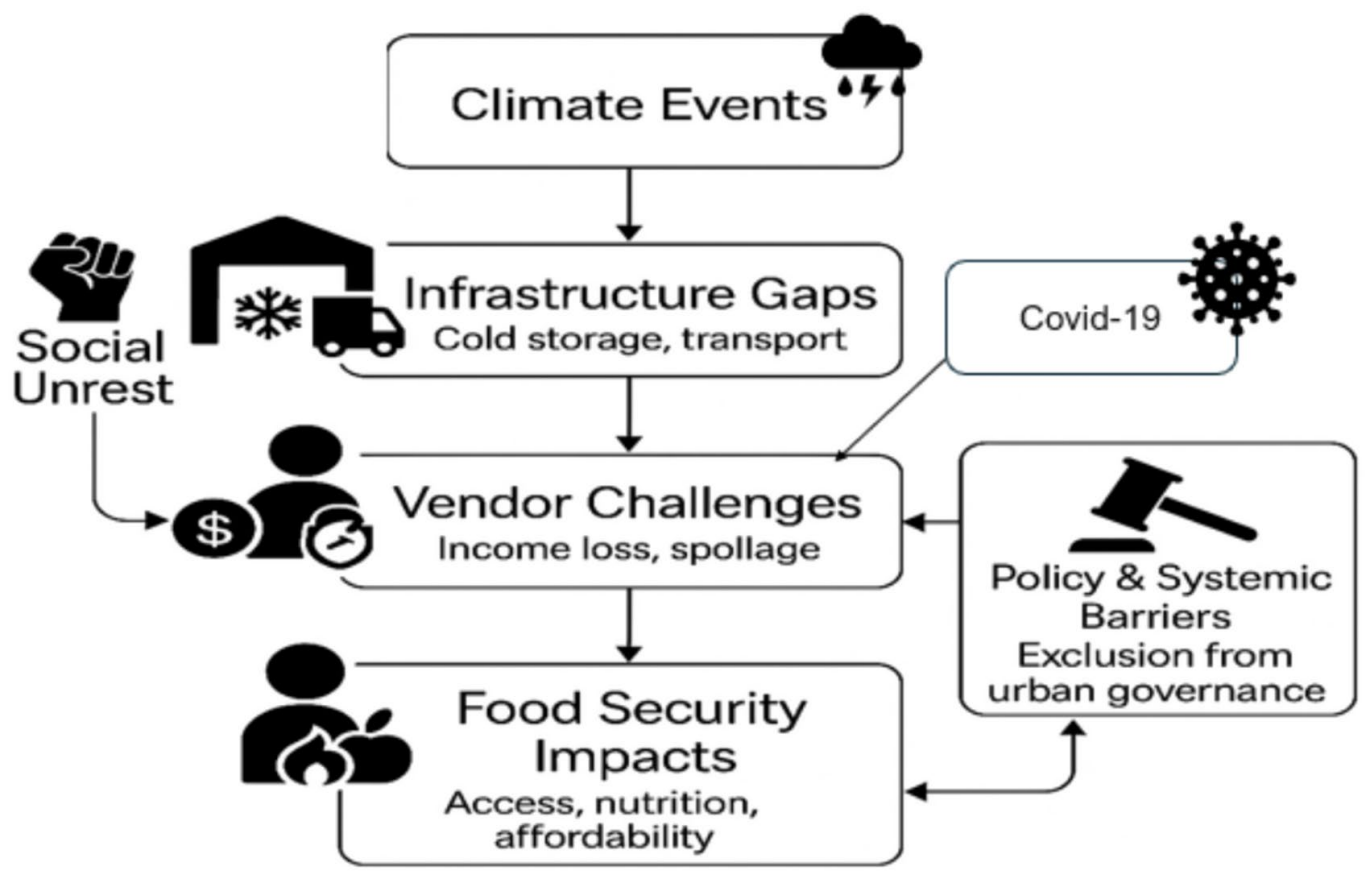
Conceptual model illustrating the systemic linkages between climate shocks, infrastructure gaps, vendor challenges, and food security outcomes (Source: Author’s own).

Built upon theoretical foundations in informality, urban risk, and food governance, the model synthesizes the interplay between exogenous shocks such as climate change, COVID-19, and social unrest and endogenous structural barriers, including infrastructure limitations and governance exclusion. As illustrated in the framework (Figure 1), climate events (e.g., floods), pandemic disruptions, and social unrest (e.g., the 2021 KZN riots) act as converging stressors that destabilize informal market systems. These shocks disrupt supply chains and intensify infrastructure gaps, such as the absence of cold storage, unreliable transport, and informal trading spaces. These systemic deficiencies, in turn, exacerbate vendor-level challenges, notably income loss, spoilage of perishable indigenous crops, and mounting fixed costs.

Moreover, the framework highlights the bidirectional relationship between vendor challenges and policy/systemic barriers. Vendors are often excluded from infrastructure development, emergency response systems, and food governance dialogs thus limiting their capacity to adapt or recover. Simultaneously, worsening vendor vulnerability reinforces their marginal status, perpetuating a cycle of neglect in policy planning and resource allocation. The final node food security outcomes capture the downstream impacts on access, nutrition, and affordability. As vendors struggle to maintain income and stock, urban consumers lose access to culturally relevant, nutritious indigenous crops. The framework thus situates informal vendors not only as economic actors but also as frontline agents in urban food resilience. This model offers a systems-level perspective aligning with recent calls for integrative urban food governance. It moves beyond single-shock models by considering how multiple, compounding stressors intersect with existing inequalities, providing a more realistic lens to understand the fragility of informal food networks in cities like Durban.

### 2.2 Nutritional and climate-smart value of indigenous crops

Indigenous food crops such as *Amaranthus* spp. (imbuya), *Colocasia esculenta* (amadumbe), and *Cucurbita maxima* (imfino yezintanga) are nutritionally dense and culturally significant, offering essential micronutrients, fiber, and plant-based protein (28, 29). Their adaptability to drought and poor soils also positions them as key assets for climate-smart agriculture in low-resource settings (28). Despite these benefits, they remain marginalized in formal food systems due to limited policy support, market visibility, and changing consumer preferences (9, 10).

Their exclusion from national nutrition strategies and institutional markets has led to persistent underutilization, even in urban areas where dietary diversity is critical. Most studies focus on producers or formal retail chains, with limited attention to informal vendors who act as primary distributors in cities (30). This gap in literature weakens understanding of how indigenous crops can support both nutrition and urban resilience. Supporting their integration across the value chain, particularly through vendor empowerment, remains essential for sustainable urban food systems.

### 2.3 Value chain constraints: post-harvest, storage, and processing

One of the most critical barriers is the lack of cold storage and proper handling infrastructure, which leads to high post-harvest losses, reduced profit margins, and frequent spoilage (18). Post-harvest losses are a recurrent issue, particularly for leafy greens such as imbuya and imfino yezintanga, which have short shelf lives and require specialized handling. Research by (28, 31, 32) found that the absence of cold storage facilities and appropriate packaging increases spoilage, decreases vendor income, and limits the availability of these crops in urban food systems. The situation is exacerbated by the lack of agro-processing skills, which could enable vendors to add value and extend product shelf life and offer various products to the market (33). Vendors are often forced to sell highly perishable produce at low prices to avoid complete loss and undermine both food quality and financial sustainability (33).

### 2.4 Urban infrastructure, climate vulnerability, and disaster risks

The broader urban food environment in South Africa presents additional barriers. Indigenous crop vendors operate in under-regulated spaces, often excluded from urban planning, disaster response, and infrastructure investments (30). Informal vendors lack formal recognition and are regularly sidelined in policy decisions, which undermines their ability to access markets, finance, and institutional support (4). Climate change compounds these vulnerabilities: unpredictable rainfall, floods, and rising temperatures disrupt transportation, market operations, and crop availability heightening food insecurity and undermining vendor resilience (12, 28).

### 2.5 COVID-19 and informal market fragility

In 2020, the COVID-19 pandemic exposed the fragility of the informal sector, where prolonged lockdowns and restricted movement disproportionately impacted small-scale traders (34). Another challenge is the absence of formal recognition and investment in indigenous crop markets. Vendors rely on informal networks for crop sourcing and sales, operate in under-regulated spaces, and often lack access to training in food safety, financial management, and agro-processing (33). Capacity development or institutional inclusion, and market integration of indigenous crop vendors, exposes them to remain vulnerable to economic shocks and further excludes them from urban food system governance (35).

### 2.6 Income loss comparisons and research gap

While extensive research has examined income loss due to climate and market shocks among commercial and smallholder farmers of staple crops like maize (36) and potatoes (37), there is a notable absence of studies focused on indigenous crop street vendors. For example (12), used an integrated assessment model to show that maize yields in South Africa’s Free State Province could decline by 10–16%, resulting in significant income reductions under climate stress scenarios. In Kenya (38), found that 19% of potato produce was lost post-harvest, representing a loss of approximately US$ 109 million annually, an income shock for smallholder growers. Similarly (39), reported that Ethiopian potato farmers experienced post-harvest losses of 21.7 kg per quintal (~36% of gross margin), significantly reducing farm income and profitability. While these studies provide valuable insights into the economic vulnerabilities of staple crop producers, their focus remains on formal agricultural systems and rural smallholders. Much less is known about the volume and impact of income loss among informal, urban street vendors selling indigenous and highly perishable crops, whose market dynamics and resilience strategies differ substantially from those of maize and potato farmers.

### 2.7 The case of Durban: context and gaps

Durban’s vulnerability to climate events such as the 2022 floods and the 2021 political unrest illustrates how external shocks weaken the entire value chain, from farm to market stall (4). During these crises, vendors experienced infrastructure damage, theft, and loss of goods, as informal markets are rarely included in disaster planning and recovery frameworks (4). These events plunged the city into greater chaos, straining its financial resources, reducing economic activity, whilst this minimized consistent trade for street vendors. Therefore, this study addresses a critical gap in urban food systems research by examining the lived experiences of 34 indigenous food crop street vendors operating in Durban’s Early Morning Market. Specifically, it investigates how climate-related shocks, infrastructure limitations, and policy gaps constrain their market participation, with a focus on six core crops commonly traded in the region. This study adopts a systems-thinking approach to explore the intersectionality of shocks (climate, social unrest, pandemics), vendor resilience, and institutional exclusion. Although limited in sample size, the study provides novel insights into urban food vulnerability from the bottom up, shedding light on the real-time coping strategies employed by informal vendors to sustain culturally relevant food systems.

## 3 Methods and materials

### 3.1 Study area

**Figure 2:**
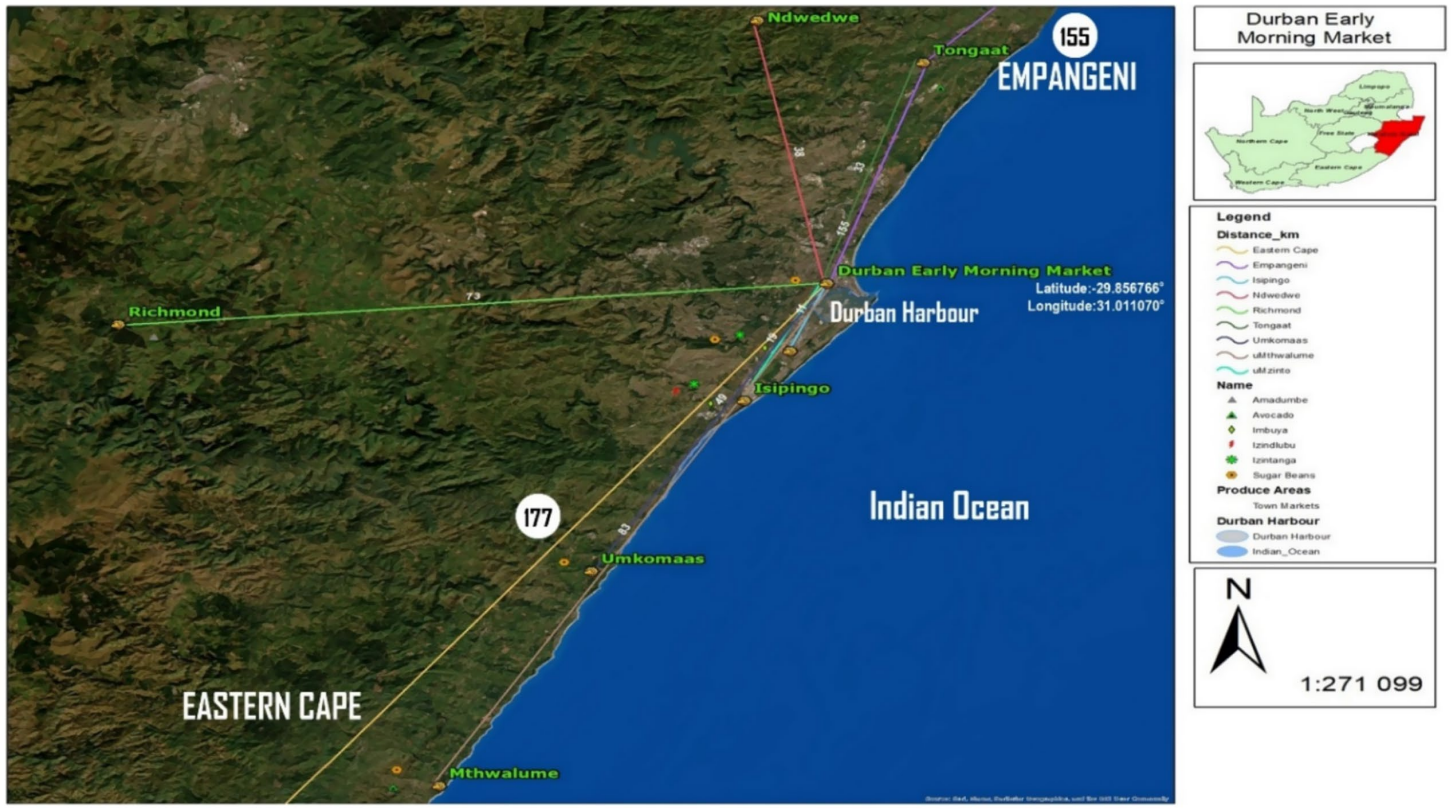
Study area showing the Durban early morning market and associated indigenous crop producer regions in KwaZulu-Natal, South Africa (Source: (75)).

This study was conducted in Durban, located within the KwaZulu-Natal (KZN) province of South Africa (Figure 2). Durban is South Africa’s third-largest city and serves as a strategic economic and trade hub due to its status as home to the country’s busiest port, the Durban Harbor (40). This positioning makes it a pivotal gateway for both domestic and international trade. The city is demographically diverse and marked by a dual food economy in which formal and informal food systems coexist, enabling access to food across various socioeconomic groups (4). Durban’s urban landscape is undergoing rapid transformation driven by urbanization and population growth (4). These dynamics have intensified the demand for affordable, culturally relevant foods, thereby reinforcing the importance of urban markets (4). Within this context, Indigenous Food Crop (IFC) vendors play a vital role in sustaining household food security, particularly for low-income urban dwellers. These areas serve as key producer zones for a variety of indigenous food crops such as taro roots, sweetpotato and many more. The location of Durban as a coastal city on the east coast of South Africa is highly susceptible to climate change-related risks, hence, Figure 1 depicts how it lies in the Indian Ocean coastal margin. Recently, eThekwini has been experiencing a climatic shift with an increase in sea level rise, high temperatures and coastal flooding. Different climate modeling tools have shown that Durban will have an elevation of temperature ranges from 0.8 °C and 1.6 °C by 2050 (41).

### 3.2 Research design and sampling strategy

This study adopted a quantitative approach to assess the effects of climate variability and market disruptions on the income, infrastructure access, and market participation of indigenous crop street vendors in Durban, South Africa. Structured questionnaires were used to collect numeric data on vendors’ socio-economic characteristics, crop perishability, storage access, and experiences with environmental and socio-political shocks. A purposive sampling strategy was employed to select all 34 formally registered indigenous food crop vendors operating at the Warwick Early Morning Market constituting a complete census of this bounded and underrepresented population. This approach was verified in consultation with market authorities and the vendor committee. Participants were eligible if they actively sold at least one of six prioritized indigenous crops: *amadumbe*, *ubhatata*, *imbuya*, *imfino yezintanga*, *intshungu*, or *amantongomane*, selected for their cultural relevance, nutritional value, and high perishability. While alternative sampling strategies (e.g., random or snowball sampling) could have included unregistered or peripheral vendors, purposive sampling was most appropriate given the study’s specific focus. This method ensured internal validity by gathering context-specific insights from vendors directly impacted by climatic and market shocks.

While the sample size of 34 may appear small, it represents the entire population of formally registered indigenous crop vendors at the Warwick Early Morning Market, making the study a complete census of this specific subgroup. This approach enhances the internal validity of the findings by eliminating sampling error and ensuring comprehensive representation of the target population. Additionally, robust statistical techniques were employed to address issues commonly associated with small samples, such as outliers and non-normal distributions. Although the findings may not be generalizable to all informal vendors in Durban or beyond, they provide reliable, context-specific insights for this underrepresented and policy-relevant group.

### 3.3 Data collection procedures

Data were collected through structured, paper-based questionnaires administered in person to street vendors at the Warwick Early Morning Market in Durban. The survey instrument was designed to capture quantitative data on vendors’ demographic characteristics, types of indigenous crops sold, income levels, storage access, and exposure to environmental and socio-political disruptions, including floods, droughts, and social unrest. The income loss data were self-reported using structured recall questions triangulated with fixed cost estimations (e.g., storage and rental fees) to mitigate recall bias. Enumerators probed for consistency across shock events to ensure reliability.

The questionnaire was developed in English and translated orally where necessary during administration to accommodate participants whose primary language was isiZulu. Data collection took place over a two-week period in April 2023. The researchers visited the market during business hours and conducted surveys face-to-face, ensuring clarity of questions and completeness of responses. Participation was voluntary, and written informed consent was obtained from all respondents. The research team adhered to ethical protocols approved by the institutional ethics committee. Completed questionnaires were reviewed daily for consistency and were later digitized and entered a secure spreadsheet for analysis.

### 3.4 Data analysis

Quantitative data collected through structured questionnaires were first entered into Microsoft Excel and subsequently exported to Stata Version 18 for analysis. The analysis included both descriptive and inferential statistical techniques, aimed at exploring vendor demographics, business characteristics, and vulnerability to climate and market shocks.

Descriptive statistics such as means, standard deviations, frequencies, percentages, minimum, and maximum values were used to summarize key attributes of the vendor population (*n* = 34). These included socio-economic profiles, types of crops sold, access to infrastructure, and income losses resulting from recent shocks such as COVID-19, the 2021 civil unrest, and flooding events. This analysis provided a comprehensive overview of patterns and variability within the dataset. To examine the determinants of income loss among vendors, a robust regression model was employed. This method was selected after the Breusch–Pagan test indicated the presence of heteroskedasticity in the dataset (*χ*^2^ = 28.78; *p* < 0.001) as shown in Table 1, violating the assumptions of classical linear regression. Robust regression corrects for heteroskedasticity by adjusting the standard errors, thereby providing more accurate and reliable coefficient estimates, even in the presence of outliers or non-constant error variance.

**Table 1:** Heteroskedasticity test (Breusch-Pagan) (Source: Author’s own).

Test	Chi^2^ (1)	*p*-value
Breusch-Pagan test	28.78	0.0000

#### 3.4.1 Empirical model specification: robust regression model

This study employs a robust linear regression model to investigate the determinants of income loss among street vendors selling indigenous food crops in the eThekwini Metropolitan District. The choice of robust regression (*rreg*) is both methodologically and empirically justified. Preliminary diagnostic tests revealed violations of key ordinary least squares (OLS) assumptions, including the presence of heteroskedasticity (Breusch–Pagan/Cook–Weisberg test, *p* < 0.05) and influential outliers, which are common in small-sample, survey-based data collected from informal vendors. OLS is known to be sensitive to such violations, leading to inefficient and biased parameter estimates. In contrast, robust regression employs an iteratively reweighted least squares (IRLS) approach that minimizes the influence of extreme residuals. It begins with Huber weights to reduce the leverage of moderately deviant observations and progresses to Tukey’s biweight function to suppress the impact of extreme outliers, resulting in more stable and reliable estimates.

Although the sample size of 34 is small, it constitutes a complete census of formally registered indigenous crop vendors in the Warwick Early Morning Market. Given this bounded population and the presence of categorical predictors, we employed a robust regression model to account for potential non-normality and outliers, which are common in small datasets from informal sector research. Robust regression down-weights extreme values and provides more reliable coefficient estimates than ordinary least squares in such conditions. As shown in (42), who applied robust regression to two small-sample neuroscience datasets (*n* = 37 and *n* = 46), the method yielded stable and conservative results when classical assumptions were violated. They further argue that the use of parametric models is statistically valid for sample sizes ≥ 30 based on the Central Limit Theorem (CLT). With our sample of *n* = 34, this principle supports the appropriateness of a parametric approach. Furthermore (43), demonstrated via Monte Carlo simulations that robust regression performs consistently well in small samples (*n* = 6 to 20), especially where outliers are present. Collectively, these methodological precedents validate the use of robust regression in this study.

The use of this method is further supported by recent studies in agricultural economics. For example (44), applied robust estimation techniques to model farm income among cassava farmers in Nigeria, concluding that robust regression outperformed OLS in the presence of data irregularities. Similarly (45), used robust regression to identify factors influencing household income among smallholder farmers, demonstrating that education level was a stronger determinant of income than pesticide usage itself, highlighting the method’s utility in agricultural livelihood research. These precedents highlight that robust regression is well-suited for analyzing income data within informal, climate-sensitive agricultural markets such as those observed in this study.

The econometric model is formally specified as follows (Equation 1):


(1) $$\begin{array}{l} \text{TotalIncomeLossinco}=\beta 0+\beta 1\;\text{Genderi}+\beta 2\;\text{YearsInBusinessi}\\ +\beta 3\;\text{HouseholdSizei}+\beta 4\;\text{AgeCategoryi}+\beta 5\;\text{EducationLeveli}\\ +\beta 6\;\text{MarketRenti}+\beta 7\;\text{COVIDImpacti}+\beta 8\;\text{LootingImpacti}\\ +\beta 9\;\text{FloodImpacti}+\beta 10\;\text{StorageAccessi}+\beta 11\;\text{Perishabilityi}\\ +\beta 12\;\text{LowDemandi}+\beta 13\;\text{QualityDeclinei}+\mathrm{\varepsilon i}\; \end{array}$$

Where: TotalIncomeLossᵢ is the income loss (ZAR) reported by vendor i . The independent variables are defined, measured, and explained in detail in Table 2, which includes socio-demographic factors, structural constraints, shock exposure variables, and market/product risks. The term εᵢ denotes the error term capturing unobserved heterogeneity.

**Table 2:** Variable definitions, measurement scales, hypothesized impact on income loss, and justification for inclusion (Source: Author’s own).

Variable name	Description	Data type: measurement	Expected impact on income loss	Rationale
Gender	Vendor’s self-identified gender	Categorical: 0 = Men, 1 = Women	+ (Women may face higher losses)	Women often have fewer financial buffers and higher caregiving burdens.
Age category	Vendor’s age group	Ordinal: 1 = <35, 2 = 36–50, 3 = > 50	U-shaped (Younger/older may lose more)	Younger vendors lack experience; older vendors may face physical and market access limits.
Level of education	Highest level of education attained	Ordinal: 1 = None to 4 = post-matric	– (Higher education reduces losses)	Education enhances business literacy and climate adaptation strategies.
Household size	Number of household members supported by vendor	Continuous (number of people)	+ (More dependents = higher pressure)	Larger households divert income away from business, increasing vulnerability.
Years In business	Number of years vendor has been trading	Continuous (years)	–(Experience reduces losses)	Experience builds resilience through market knowledge and social capital.
Renting in the market	Whether vendor rents space in a formal market	Binary: 0 = No, 1 = Yes	+ (Renters face higher losses)	Fixed costs reduce flexibility and amplify losses during demand shocks.
COVID-19 impact	Whether vendor attributes income loss to COVID-19 disruptions	Binary: 0 = No, 1 = Yes	+ (COVID increased losses)	Lockdowns reduced customer access and disrupted informal supply chains.
Looting impact	Whether income loss is linked to looting or unrest	Binary: 0 = No, 1 = Yes	+ (Direct losses from looting)	Civil unrest destroyed stock, equipment, and disrupted informal business activity.
Flooding impact	Whether income loss is due to flooding or heavy rains	Binary: 0 = No, 1 = Yes	+ (Flooding increased losses)	Floods damage infrastructure, spoil produce, and block customer access.
Access/limited storage	Whether vendor has access to storage facilities	Binary: 0 = No, 1 = Yes	– (Storage reduces losses)	Secure storage prevents spoilage and enables bulk buying.
They spoil fast	Vendor reports products spoil quickly	Binary: 0 = No, 1 = Yes	+ (Spoilage = higher losses)	Perishable indigenous crops are climate-sensitive and decay fast without cold storage.
Customers buy small quantity	Vendor reports reduced customer demand or buying power	Binary: 0 = No, 1 = Yes	+ (Low demand reduces income)	Economic downturns reduce demand for informal and niche food products.
Their quality has decreased	Vendor perceives that product quality has deteriorated	Binary: 0 = No, 1 = Yes	+ (Poor quality reduces prices)	Climate stress and long supply chains may reduce crop quality, lowering willingness to pay.

The robust regression estimates the model by minimizing the weighted residual sum of squares (Equation 2):


(2) min∑i=1nwi(Yi−^Yi)2

Where:

-
$Y_{i}$ : The observed value of the dependent variable (Total income loss) for observation i .
-
^Yi : The predicted value from the regression model for observation i .
-
(Yi−^Yi)2 : The squared residual, or error term, for each observation.
-
$w_{i}$ : The weight assigned to each observation’s residual. These weights depend on how extreme the residual is.
-
$\sum_{i=1}^{n}:$ The sum across all *n* observations.
-
min : The goal is to minimize the entire sum — that is, the regression algorithm finds coefficient estimates (βs) that result in the smallest weighted sum of squared residuals.

This approach preserves all observations while reducing the influence of outliers and ensures more reliable coefficient and standard error estimates, especially in small-sample, non-normal, or heteroskedastic datasets.

While the model includes a significant number of categorical and binary variables, this reflects the socio-economic realities of the vendor population—where characteristics such as education level, crop type, access to storage, and exposure to shocks are inherently categorical. These variables are essential for capturing the multidimensional factors influencing income loss and market participation. To address potential multicollinearity, dummy variables were constructed with appropriate reference categories, and model specification was carefully examined. Variance Inflation Factor (VIF) analysis was conducted, and no predictor exceeded the conventional threshold of 10, indicating no serious multicollinearity concerns. The use of robust regression further supports the reliability of the findings, as it accommodates violations of classical assumptions associated with small sample sizes and categorical predictors. Although the inclusion of multiple categorical variables introduces some interpretive complexity, the model yields directionally consistent and contextually meaningful insights into the socio-economic drivers affecting indigenous vendors in the post-shock environment.

### 3.5 Ethical considerations

Ethical approval (RD1/07/2023) for this study was obtained from the Mangosuthu University of Technology (MUT). Permission to conduct the study was also granted by the eThekwini Metropolitan Municipality. Prior to participation, all vendors were informed in isiZulu their primary language about the study’s objectives, data collection procedures, their right to withdraw at any point, and how their data would be used. Informed verbal and written consent were obtained and appropriately documented prior to participation. Confidentiality and privacy were maintained throughout, and enumerators received training on ethical conduct.

## 4 Results

### 4.1 Demographic and socioeconomic profile of street vendors

Table 3 presents the demographic and contextual characteristics of the 34 indigenous food crop street vendors surveyed in Durban’s Early Morning Market. The vendor population was predominantly female (85.3%, *n* = 29), reflecting the gendered nature of informal food vending in urban South Africa. The majority of participants (44.1%) were aged between 36 and 50 years, followed by those under 35 (32.4%) and over 50 (23.5%). Educational attainment was relatively high for an informal sector cohort: 76.5% had completed high school, while a small number had post-matric qualifications (5.9%).

**Table 3:** Socio-demographic and market shock characteristics of indigenous crop street vendors (Source: Author’s own).

Category	Subcategory	Percentage %	Frequency (*n*)
Gender	Women	85.3	29
	Men	14.7	5
Age category	<35 years	32.4	11
	36–50 years	44.1	15
	>50 years	23.5	8
Level of education	No school background	2.9	1
	Primary school	14.7	5
	High school (up to Matric)	76.5	26
	Post-matric (Diploma/Certificate)	5.9	2
Renting in the market	0 = No	17.65	6
	1 = Yes	82.35	28
COVID-19 impact	0 = No	47.06	16
	1 = Yes	52.94	18
Looting impact	0 = No	35.29	12
	1 = Yes	64.71	22
Flooding impact	0 = No	38.24	13
	1 = Yes	61.76	21
Access/limited storage	0 = No	47.06	16
	1 = Yes	52.94	18
They spoil fast	0 = No	17.65	6
	1 = Yes	82.35	28
Customers buy small quantity	0 = No	55.88	19
	1 = Yes	44.12	15
Their quality has decreased	0 = No	47.06	16
	1 = Yes	52.94	18

Regarding market dynamics, a substantial majority of vendors (82.35%) reported renting space within the market, indicating a structured relationship with the trading environment. Over half of the respondents (52.94%) reported being negatively affected by COVID-19 lockdowns, while 64.71 and 61.76% reported income disruptions from the 2021 KZN unrest and the 2022 flooding event, respectively. This confirms the significant exposure of informal traders to socio-environmental and economic shocks.

In terms of market constraints, a substantial majority of respondents (82.35%) indicated that indigenous crops spoil quickly, highlighting spoilage as a significant post-harvest challenge. This finding suggests that product perishability is a major vulnerability, likely linked to limited cold storage infrastructure and inadequate handling practices. Additionally, over half of the vendors reported experiencing limited storage access (52.94%) and a decline in product quality (52.94%), reinforcing the urgent need for improved post-harvest systems. Around 44.12% observed that customers buy in small quantities, a factor that may reduce turnover and contribute to cumulative income loss.

Table 4 summarizes key continuous variables. The mean total income loss experienced across the three major shocks (COVID-19, unrest, floods) was ZAR 13,111.76, with a high standard deviation (ZAR 13,643.80), reflecting considerable variation in impact across vendors. The average household size was large (≈8 members), consistent with broader trends in low-income urban communities. Vendors had an average of 12.1 years of business experience, with some reporting up to 31 years in the sector, indicating a highly experienced cohort navigating persistent systemic challenges.

**Table 4:** Summary statistics for key continuous variables: income loss, household size, and business experience (Source: Author’s own).

Variable	Mean	Std. dev.	Min	Max
Total income loss	13111.76	13643.8	500	70,000
No. of household members	7.970588	3.05986	4	15
Years of business	12.11765	9.047902	2	31

### 4.2 Impact of market shocks on average vendor income loss

**Figure 3:**
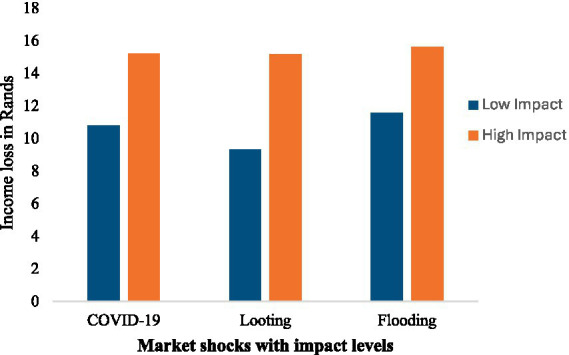
Impact of market shocks on average vendor income loss.

Figure 3 presents the average income loss in Rands experienced by vendors in response to three market shocks such as COVID-19, looting, and flooding, each classified by low and high impact levels. The results show a clear pattern: across all shock types, vendors who reported high levels of impact experienced significantly greater income losses compared to those who experienced low impact. For COVID-19, the average income loss among vendors with high impact was approximately R15,200, compared to around R10,800 for those with low impact. A similar trend is evident in the context of looting, where high-impact vendors lost about R15,200 on average, while low-impact vendors lost around R9,300. In the case of flooding, the average loss for high-impact vendors was approximately R15,600, compared to R11,600 for those who reported lower impact. These findings highlight the strong relationship between the severity of external shocks and financial vulnerability among informal street vendors. They reinforce the need for targeted interventions, such as microgrant schemes, infrastructure improvements, and inclusive disaster risk planning, to mitigate income volatility and enhance resilience in the informal food sector.

### 4.3 Financial pressures behind the stall: storage, transport, and rent

Table 5 outlines the core fixed operational expenses incurred by indigenous food crop street vendors. The annual stall rental fee is relatively low at ZAR 530 (≈USD 28.19), reflecting the informal nature and subsidized access associated with the Early Morning Market. However, ongoing operational costs accumulate significantly over time. Vendors pay ZAR 50 (USD 2.66) weekly for weekday storage and ZAR 40 (USD 2.13) for weekend storage, highlighting the importance of cold storage access as both a logistical and financial burden. Additionally, the daily cost of hiring a street porter to transport goods is ZAR 50 (USD 2.66), a necessity for vendors managing bulk produce without private vehicles or storage access.

**Table 5:** Fixed operational costs (Source: Author’s own).

Item	Amount (ZAR)	Equivalent (USD)	Frequency
Stall rental	530	$28.19	Annually
Storage rental (weekday)	50	$2.66	Weekly
Storage rental (weekend)	40	$2.13	Per weekend
Street porter	50	$2.66	Daily

### 4.4 Synopsis of indigenous crop diversity, value chain dynamics, and product offerings among Durban market street vendors

Tables 6, 7 presents an integrated overview of six key indigenous food crops sold by street vendors in Durban’s informal market system. The analysis highlights variation in vendor participation rates, price ranges, packaging norms, post-harvest handling practices, and consumer utilization patterns.

**Table 6:** Sales and packaging of indigenous crops sold by street vendors in Durban (Source: Author’s own).

Crop	% Vendors	Avg. price (ZAR)	Packaging	Peak sales
Ubhatata (Sweetpotato)	70.6%	40–360	50 kg bags	Weekends (41.2%)
Amadumbe (Taro)	64.7%	35–400	50 kg bags	Weekends (38.2%)
Imfino yezintanga (Pumpkin Leaves)	38.2%	10–30	4-bunch bundles	Weekdays (23.5%)
Imbuya (Pigweed)	29.4%	5–30	Small/medium bags	Weekends (17.6%)
Umdumbulu (Cassava)	5.9%	20–30	Loose/kg	Weekends (5.9%)
Amantongomane (Groundnuts)	8.8%	10–35	10-unit batches	Irregular

**Table 7:** Sourcing, handling, use and nutrients (Source: Author’s own).

Crop	Source and value chain	Post-Harvest handling	Consumer use	Nutrient highlights
Ubhatata (Sweetpotato)	Rural farmers; sold via middlemen	Dry storage; prone to sprouting	Boiled, roasted, mashed	CHO 20.1 g; Vit C 23 mg; Energy 385 kJ (76, 77)
Amadumbe (Taro roots)	Highlands of KZN; sold through traders	Crates; susceptible to mold	Boiled staple starch	CHO 28.9 g; Ca 22 mg; Energy 439 kJ (78)
Imfino yezintanga (Pumpkin Leaves)	Rural producers or self-grown	Damp paper wrapping; highly perishable	Cooked as greens	CHO 21.3 g; Ca 28 mg; Protein 1.7 g (79)
Imbuya (Pigweed)	Rural areas; home gardens	No cold chain; spoils fast	Traditional relish	Protein 2.5 g; Fe 8.9 mg; Vit A 850 IU (80)
Umdumbulu (Cassava)	Smallholder farmers	Perishable; unprocessed	Boiled or steamed	CHO 38 g; Vit C 25 mg (81)
Amantongomane (Groundnuts)	Farmers and wholesalers	Dry storage; long shelf life	Snack, paste, stew	Protein 25.8 g; Fat 38.3 g; Energy-rich (46)

CHO, Carbohydrate; TFib, Total Fiber; Prot, Protein; Ca, Calcium; Fe, Iron. ZAR to USD exchange rate used: 1 USD ≈ ZAR 18.80 (April 2025). Peak sales reflect vendor-indicated high-demand days. Nutrient values drawn from standard African indigenous crop databases. *Street Shelf-life A (High risk to extreme heat temperatures- 1-day high losses). **Street Shelf-life B (Medium risk – 5-7 days, minimal losses often include weekend purchases where sales are high). ***Street Shelf-life C (Long-term risk 1- months dry maximized sales over duration storage period).

#### 4.4.1 Vendor participation and crop popularity

As shown in Table 6 Ubhatata (*Ipomoea batatas*, sweetpotato) emerged as the most widely traded crop, sold by 70.6% of vendors. This was closely followed by amadumbe (*Colocasia esculenta*, taro) at 64.7%. Traditional green leafy vegetables such as imfino yezintanga (*Cucurbita maxima* leaves) and imbuya (*Amaranthus* spp.) were sold by 38.2 and 29.4% of vendors, respectively. Less frequently sold crops included umdumbulu (*Manihot esculenta*, cassava) at 5.9% and amantongomane (*Arachis hypogaea*, groundnuts) at 8.8%, reflecting their more specialized or seasonal demand.

#### 4.4.2 Pricing and packaging practices

Significant price variation was observed across crops. Ubhatata and amadumbe recorded the highest price ranges, R40–360 (US$2.09–18.82) and R35–400 (US$1.83–20.91) respectively, and were generally sold in 50 kg bags, indicating bulk trading. Imfino yezintanga and imbuya were more affordably priced between R5–30 (US$0.26–1.57) and typically packaged in small bundles or bags, enabling access for low-income consumers. Umdumbulu and amantongomane were sold by weight or in pre-measured batches, offering greater flexibility in pricing and packaging.

#### 4.4.3 Sales patterns

Table 6 indicates that weekend trading dominated peak sales periods for most crops, particularly ubhatata (41.2%) and amadumbe (38.2%), suggesting alignment with consumer purchasing cycles and market foot traffic. Imfino and imbuya had modest weekday sales, while cassava and groundnuts were sold irregularly, indicating sporadic availability or lower mainstream demand.

#### 4.4.4 Sourcing and value chain linkages

Sourcing varied depending on the crop. Ubhatata and amadumbe were largely sourced from rural farmers and highland regions via traders, reflecting relatively extended value chains. Imfino and imbuya were mostly grown by vendors themselves or acquired from local rural suppliers (as shown in Table 7), demonstrating short, informal supply loops. Cassava was obtained from smallholder farmers, while groundnuts were accessed from both farmers and wholesalers, implying wider sourcing networks.

#### 4.4.5 Post-harvest handling

Handling methods reflected the perishability of each crop. Imfino and imbuya lacked cold chain support and were prone to spoilage, despite being wrapped in damp material. Amadumbe required crate storage to avoid mold, while ubhatata maintained shelf life under dry conditions but could sprout over time. Cassava remained perishable and largely unprocessed, while groundnuts benefited from dry storage and long shelf stability, making them a low-risk commodity for vendors.

#### 4.4.6 Consumer use and nutritional attributes

Consumer usage aligned with local dietary norms. Root crops like ubhatata, amadumbe, and umdumbulu were used as staple starches, commonly boiled or mashed. Imfino and imbuya were used in traditional vegetable dishes or relishes, maintaining cultural culinary relevance (see Table 7). Amantongomane (groundnuts) were consumed as snacks, added to stews, or processed into paste. Nutritional profiles emphasized the value of these crops: imbuya was particularly high in iron (8.9 mg) and vitamin A (850 IU), while groundnuts provided dense levels of protein (25.8 g) and fat (38.3 g), making them essential contributors to energy and nutrient intake (46).

### 4.5 Econometric results

#### 4.5.1 Multicollinearity check

Table 8 reports the Variance Inflation Factors (VIFs) for all explanatory variables to assess multicollinearity. All VIF values were below the conventional threshold of 10, with the highest being 3.09 (for Age Category). The mean VIF was 1.87, indicating a low level of multicollinearity among the predictor variables. This suggests that the regression coefficients are not distorted due to strong correlations among independent variables.

**Table 8:** Variance inflation factor (VIF) for multicollinearity check (Source: Author’s own).

Variable	VIF	1/VIF
Age category	3.09	0.324
Years of business	2.23	0.448
Their quality has decreased	2.16	0.463
Access/limited storage	1.89	0.530
Customers buy small quantity	1.83	0.547
No. of household members	1.82	0.550
COVID impact	1.81	0.552
Gender	1.75	0.571
Renting in the market	1.75	0.572
Looting impact	1.71	0.586
Highest education qualification	1.63	0.615
Flooding impact	1.36	0.738
They spoil fast	1.33	0.752
Mean VIF	1.87	

#### 4.5.2 Determinants of income loss among indigenous crop vendors: robust regression results

The robust regression analysis presented in Table 9 examines the influence of various demographic factors, market shocks, and structural constraints on the total income loss experienced by indigenous crop street vendors following the COVID-19 pandemic, the 2021 KwaZulu-Natal unrest, and the 2022 floods. The model explains approximately 51.6% of the variation in income loss (R-squared = 0.5160), indicating a good fit. Several variables, including education level, age category, and renting in the market, show statistically significant effects on income loss among vendors.

**Table 9:** Determinants of income loss among indigenous crop vendors (Source: Author’s own).

Variable	Coef.	Std. Err	*t*-value	*p*-value	95% CI
Gender	−2,970.91	1,711.35	−1.74	0.098*	(−6,541, 599)
Years of business	−52.84	76.74	−0.69	0.499	(−213, 107)
No. of household members	−699.43	204.89	−3.41	0.003***	(−1,127, −272)
Age category	2,556.48	691.76	3.70	0.001***	(1,113, 3,999)
Level of education	4,827.49	673.84	7.16	0.000***	(3,422, 6,233)
Renting in the market	−11,696.40	1,588.40	−7.36	0.000***	(−15,010, −8,383)
COVID impact	4,045.47	1,234.78	3.28	0.004***	(1,470, 6,621)
Looting impact	−1,025.92	1,252.73	−0.82	0.422	(−3,639, 1,587)
Flooding impact	3,986.45	1,097.64	3.63	0.002***	(6,276, 1,697)
Access/limited storage	2,501.41	1,260.33	1.98	0.061*	(−128, 5,130)
They spoil fast	3,651.53	1,385.89	2.63	0.016**	(761, 6,842)
Customers buy small quantity	51.57	1,247.97	0.04	0.967	(−2,252, 2,655)
Their quality has decreased	−7,197.85	1,348.67	−5.34	0.000***	(−10,011, −4,385)
Constant (cons)	20,014.21	5,935.42	3.37	0.003***	(7,633, 32,395)

Observations (N): 34 F-statistic: 13.73 Prob>F: 0.0000 R-squared = 0.5160 Statistical significance is denoted as follows:**p < 0.01; **p < 0.05; and *p < 0.1.

The negative coefficient (−R2,970.91) suggests male vendors experienced marginally lower income losses than females, though this effect was significant only at the 10% level (*p* = 0.098). The 95% confidence interval spanning from -R6,541 to R599 includes zero, indicating uncertainty about the true effect size. The marginal significance (*p* < 0.1) warrants caution in interpretation given the small male subsample (*n* = 5). Each additional household member was associated with R699.43 higher income loss (*p* = 0.003), with the narrow 95% CI excluding zero. This implies 99% confidence that the true effect lies between -R1,127 and -R272 per member. The result mirrors findings by (47) in East Africa, where larger households exhibited greater financial fragility during COVID-19, likely due to heightened subsistence pressures.

The regression results reveal a statistically significant and positive association between education level and income loss (*β* = R4,827.49; *p* < 0.001), with a 95% confidence interval ranging from R3,422 to R6,233. Stall renters endured R11,696.40 greater losses (*p* < 0.001), with the 95% CI (−R15,010 to -R8,383) confirming high precision. This aligns with (48), where fixed costs amplified vendor vulnerability during West African food crises. The CI width (R6,627) suggests moderate variability around this large effect. COVID-19-affected vendors lost R4,045.47 more income (*p* < 0.01), with 95% confidence the true effect lies between R1,470 and R6,621. The CI’s positive range confirms the shock’s severe, unidirectional harm.

Flooding caused R3,986.45 additional losses (*p* < 0.01), with 95% CI bounds (R1,697 to R6,276) excluding zero. The narrow range (width = R4,579) indicates precise estimation, supporting (28) on climate shocks’ disproportionate effects on perishable crop vendors. Storage-constrained vendors faced R2,501.41 higher losses (*p* = 0.061), but the 95% CI (−R128 to R5,130) includes zero, suggesting marginal significance. While the point estimate aligns with (29) on post-harvest losses, the CI’s inclusion of null effects necessitates cautious interpretation. Perishability drove R3,651.53 greater losses (*p* < 0.05), with 95% CI (R761 to R6,842) confirming significance. The CI’s lower bound >0 confirms robustness. Perceived quality decline led to R7,197.85 higher losses (*p* < 0.001), with 95% CI (−R10,011 to -R4,385). The entirely negative CI strongly supports (49) on quality-price linkages in informal markets. The effect’s magnitude underscores consumer sensitivity to perceived crop integrity.

## 5 Discussion

### 5.1 Demographic and socioeconomic profile of indigenous crop street vendors

This study’s demographic profile of indigenous crop street vendors in Durban reveals a highly gendered and economically embedded informal economy shaped by structural vulnerabilities, demographic realities, and exposure to compounding socio-environmental shocks. The descriptive data shows that 85.3% of vendors were women, aligning with longstanding evidence that informal food vending in sub-Saharan Africa is predominantly a female-dominated livelihood strategy (27). This gendered trend has been similarly documented by (50), who found that urban agriculture and informal retailing offer accessible, albeit precarious, income opportunities for women, particularly those with limited formal education or capital. The vendors in this study were relatively experienced, with an average of 12.1 years in business, and belonged to diverse age cohorts—44.1% were aged 36–50, and 32.4% were younger than 35. This aligns with earlier findings from urban Ghana, where the majority of market vendors were aged above 21–50, suggesting that Durban’s market sector attracts both seasoned and younger participants (51). The relatively high educational attainment (76.5% with high school education) among these vendors is also notable and may reflect an evolution in informal market composition, potentially linked to increasing unemployment among semi-skilled individuals in South Africa (52).

A key vulnerability emerged from the finding that 82.35% of vendors rented market space, often incurring fixed operational costs. This mirrors findings from (53, 54), who observed that access to formal trading space often comes with increased exposure to financial risk during market or climate-related disruptions. These financial commitments, when combined with frequent external shocks, create precarious income patterns. Indeed, over half of the respondents in this study reported income losses due to COVID-19 (52.94%), flooding (61.76%), and the 2021 unrest (64.71%), consistent with regional analyses that emphasize the vulnerability of informal vendors to compound disasters (55).

Post-harvest challenges were a prominent theme, with 82.35% of vendors stating that their indigenous produce spoils quickly, while over half experienced limited storage access (52.94%) and declining crop quality (52.94%). These challenges are consistent with findings by (56), who attributed post-harvest losses of traditional vegetables to limited cold chain availability, poor packaging, and inadequate market infrastructure. These constraints not only diminish vendor profitability but also compromise consumer access to nutrient-dense foods. Interestingly, 44.12% of vendors reported that customers typically buy small quantities. This aligns with research by (57), who observed that affordability and perishability drive urban consumer preferences for “daily micro-purchases” rather than bulk buying, especially among low-income populations.

On average, vendors reported household sizes of ≈8 members, further highlighting the extended socio-economic dependencies supported by street vending activities. This parallels findings by (58), who found that small-scale informal trade activities in Africa frequently serve as multi-generational livelihood buffers, especially where formal employment opportunities are scarce. Finally, the mean income loss of ZAR 13,111 during combined shocks reflects significant economic hardship and aligns with similar loss magnitudes reported in post-COVID-19 assessments of smallholder and informal actors in Eastern and Southern Africa (59).

### 5.2 Operational cost structures and their implications for vendor vulnerability

#### 5.2.1 Subsidized stall rental amid accumulating hidden costs

The annual stall rental fee of ZAR 530 (≈USD 28.19) reflects a degree of institutional support and municipal subsidy that enables traders to access formal trading platforms within the Early Morning Market. Such access is vital, as it provides physical visibility, consumer foot traffic, and perceived legitimacy factors that enhance both income generation and consumer trust. However, these low entry costs may mask the high cumulative costs of daily operations, particularly for vendors with limited capital and no formal financing mechanisms. This echoes findings by (27), who emphasized that while market stall allocations and informal space tenure remain affordable, the true economic cost of informality lies in operational volatility, such as unpredictable storage access, *ad hoc* labor expenses, and supply chain disruptions.

#### 5.2.2 Cold storage and transport costs: overlooked vulnerabilities

Vendors spend ZAR 50 (USD 2.66) weekly on weekday storage and ZAR 40 (USD 2.13) over weekends, incurring monthly expenditures that quickly outstrip the annual stall rental cost. These costs are not merely financial; they reflect a systemic infrastructure gap in post-harvest preservation, which contributes directly to produce spoilage, revenue loss, and market instability. This is particularly concerning given that 82.35% of vendors reported that indigenous crops “spoil fast,” confirming that inadequate cold storage undermines the shelf life of perishable crops such as imfino and imbuya. This aligns with (60, 61), who stressed that investment in cold storage infrastructure is essential for reducing post-harvest losses and stabilizing income for small-scale traders of perishable indigenous crops. Without structural solutions, recurring storage expenses act as a tax on fragility penalizing traders who are already vulnerable to climatic and market shocks.

#### 5.2.3 Labor costs and the logistics burden

The daily fee of ZAR 50 (USD 2.66) to hire street porters highlights a common workaround among informal vendors who lack access to personal vehicles or motorized logistics. For vendors managing bulk commodities like amadumbe and ubhatata, manual transport services are indispensable but costly. Over a standard 20-day working month, this translates to ZAR 1,000 (≈USD 52.63) almost twice the annual stall rental. This aligns with urban transport and labor burden findings from (62), who found that labor costs disproportionately affect women-led food vending enterprises, due to gendered constraints on mobility and asset ownership. For women who comprise 85.3% of the vendor population in this study outsourcing heavy logistical tasks is not just convenient but necessary, yet it further diminishes net profits. Collectively, these cost dynamics expose the invisible pressures that constrain income potential and scalability within informal food systems. While indigenous crops hold nutritional and cultural value, their market success is undercut by the accumulated weight of logistical and post-harvest expenses, which are rarely accounted for in policy design. Without targeted interventions—such as subsidized storage, vendor cooperatives for shared porter services, or mobile refrigeration units—the current cost structure will continue to entrench fragility and limit the expansion of indigenous food markets.

### 5.3 Indigenous crop diversity, value chain dynamics, and market offerings

#### 5.3.1 Crop popularity and market differentiation

The most widely sold indigenous crops were Ubhatata (*Ipomoea batatas*, sweetpotato, 70.6%) and Amadumbe (*Colocasia esculenta*, taro, 64.7%), both starchy root crops that form a cornerstone of Zulu diets and offer significant caloric value. These findings resonate with those of (28) who identified root and tuber crops as crucial to dietary energy security in sub-Saharan Africa due to their adaptability, affordability, and cultural relevance. The lower market representation of crops such as Umdumbulu (cassava, 5.9%) and Amantongomane (groundnuts, 8.8%) may reflect seasonal availability constraints or supply chain fragmentation. The market’s crop diversity, while narrower than national or rural inventories, reflects consumer familiarity and preparation knowledge, which are often key drivers of urban indigenous crop consumption (63). Moreover, the sale of Imfino yezintanga (*Cucurbita maxima* leaves, 38.2%) and Imbuya (*Amaranthus* spp., 29.4%), both traditional leafy greens, confirms that street vendors remain essential custodians of indigenous vegetable biodiversity in urban settings.

#### 5.3.2 Pricing, packaging, and consumer targeting

The findings illustrate a distinct segmentation in crop pricing and packaging. High-volume, high-value crops like sweetpotato and taro were packaged in 50 kg bags, with prices ranging up to ZAR 400 (≈US$20.91), indicative of wholesale trade to households or secondary vendors. In contrast, imbuya and imfino were sold in small bags or bundles at R5–30 (≈US$0.26–1.57), targeting low-income consumers and enabling micro-purchases. This mirrors patterns reported by (11), who emphasized that informal markets excel at customizing food access in low-resource settings through flexible packaging and price structures. These pricing patterns also suggest value chain asymmetries: while bulk crops facilitate vendor economies of scale, leafy vegetables ensure consistent cash flow, albeit with higher spoilage risk. Notably, weekends were peak trading days for most vendors, especially for bulk starchy crops, reflecting a rhythm in consumer demand and market foot traffic (64).

#### 5.3.3 Post-harvest handling and shelf-life challenges

The results confirm substantial variation in post-harvest vulnerability, particularly among leafy vegetables. Imfino and imbuya were highly perishable and lacked any cold chain infrastructure, echoing warnings from (65), who identified the absence of post-harvest innovations as a leading bottleneck in the indigenous vegetable value chain. These crops were often wrapped in damp paper, which offers only short-term freshness retention and exposes the produce to fungal spoilage and nutrient loss under high temperatures. In contrast, amantongomane (groundnuts) were dry-stored and shelf-stable for months, representing a low-risk, energy-dense alternative with market longevity. Ubhatata and amadumbe, while less perishable, face sprouting or mold risks if storage conditions are inadequate. The evidence supports the argument for introducing affordable, decentralized storage technologies, such as evaporative cooling or crate-based ventilation systems, as proposed by (66).

#### 5.3.4 Nutritional significance and consumer utility

The consumer applications of these crops reflect their dual role in cultural preservation and nutritional support. Roots like cassava and sweetpotato are used as boiled staples, while leafy greens are integrated into traditional stews. Amantongomane (groundnuts) serve as both snacks and protein complements, underlining their dietary versatility. The nutrient highlights demonstrate that these crops can contribute to dietary adequacy. Imbuya, with 8.9 mg of iron and 850 IU of Vitamin A, can help combat micronutrient deficiencies, while groundnuts, with 25.8 g protein and 38.3 g fat, offer high energy density—a critical asset in food-insecure households. These findings support the advocacy by (67), who argue for elevating the policy status of indigenous foods as “nutrition-sensitive crops” that can bridge micronutrient gaps in urban poor communities. This study confirms that indigenous crops remain underutilized yet essential in urban food environments. Their diversity, affordability, and nutrient profile position them as strategic assets in building resilient, low-carbon, and culturally rooted food systems (28). However, their economic potential is constrained by infrastructural deficits, short shelf life, and narrow market reach.

### 5.4 Determinants of income loss among indigenous crop vendors

#### 5.4.1 Demographic predictors of vulnerability

The results show that female vendors experienced higher income losses than their male counterparts (*p* = 0.098), corroborating longstanding research on gender-based vulnerability in food systems. Female actors, particularly in the Global South, frequently operate at smaller scales and face disproportionate access barriers to land, capital, and formal credit, exacerbating economic exposure during crises (27). Similar findings have been reported in Zimbabwe and Malawi, where women in informal markets had less recovery capacity post-shock due to asset poverty and household care burdens.

Household size was also significantly associated with income loss (*p* = 0.003), with larger households experiencing greater vulnerability. While large households are sometimes thought to offer economic resilience through labor pooling, they may also increase food, and income demands during economic stress. This is consistent with evidence from East African urban food systems where large household sizes were linked to increased food insecurity and income volatility during COVID-19 lockdowns (47, 68).

The findings show that both age and education were positively associated with income loss may appear counterintuitive, as these factors are often linked to experience or adaptive capacity. However, in this context, they likely signal business scale and cost exposure. Although counterintuitive, the positive relationship between education and income loss may reflect that better-educated vendors manage larger operations, invest more in inventory, and rent formalized stalls exposing them to higher losses when market activity is disrupted. Thus, education may proxy business scale rather than resilience. This interpretation aligns with findings from informal markets in Lagos, where more experienced traders despite their skills reported larger absolute losses during COVID-19 due to their greater operational footprints (69). Thus, education in this setting may serve as a proxy for formalization and investment level rather than resilience per se.

#### 5.4.2 Structural constraints and fixed costs

Among the most substantial predictors was market rental, with vendors who rent stalls experiencing R11,696 more in income loss (*p* < 0.001). This reflects how fixed costs in informal systems, despite their modest absolute value, become untenable under supply shocks and revenue collapses. The importance of rental exposure is also supported by (70), who found that rental debt accumulation during COVID-19 forced many small traders in Harare into default and permanent closure.

#### 5.4.3 Market shocks: COVID-19 and flooding

The regression revealed that COVID-19 and flooding were significantly associated with elevated income loss (*p* = 0.004 and *p* = 0.002, respectively), underscoring the compounding nature of health and climate stressors on informal food systems. During the COVID-19 lockdowns, street vendors faced mobility restrictions, perishability challenges, and demand contractions. Similar disruptions were observed in Ghana and Kenya, where traders’ supply lines were fractured by travel bans, resulting in widespread spoilage and income collapse (71, 72). Flooding, as a climate-linked event, created direct barriers to market access, destroyed inventory, and damaged physical infrastructure, including informal storage spaces. These findings align with (73), who documented how flood events in informal Ugandan vegetable markets reduced vendor turnover and decimated perishables, especially among vendors lacking access to elevated or enclosed market spaces.

#### 5.4.4 Post-harvest and storage-related constraints

Post-harvest challenges were found to meaningfully shape income trajectories. Vendors who reported fast spoilage of indigenous crops incurred R3,652 more in income loss (*p* = 0.016), highlighting the vulnerability of leafy greens and unprocessed tubers. Similarly, limited access to storage was marginally significant (*p* = 0.061), but the estimated effect size suggests substantive impact. These findings resonate with (28), who argue that underutilised crops are severely constrained by lack of post-harvest innovations in sub-Saharan Africa, particularly cooling, sorting, and packaging technologies. Most notably, vendors who perceived that crop quality had decreased experienced R7,198 greater income loss (*p* < 0.001). This perception likely captures both physical deterioration due to weather variability and reduced consumer trust in product integrity. Prior work has emphasized that the subjective quality of indigenous foods affects not only pricing but also market re-entry rates for vendors post-harvest (74).

## 6 Conclusion and policy implications

This study set out to investigate the determinants of income loss among street vendors selling indigenous food crops in the eThekwini Metropolitan District, with a specific focus on understanding how socio-demographic characteristics, infrastructural access, and climate-related shocks interact to shape economic vulnerability in informal agricultural markets. Through a combination of descriptive statistics and robust regression modeling, the study successfully identified a range of factors that significantly influence income losses—ranging from gender and household size to structural constraints such as market rent, inadequate storage, and post-harvest spoilage. A key contribution of the study lies in its empirical focus on indigenous crop street vendors, an often-overlooked segment in food systems research that typically centers on commercial or smallholder farmers growing mainstream crops. By foregrounding vendors of traditional crops like *imbuya*, *amadumbe*, and *ubhatata*, the research underscores the critical role of informal trade in sustaining urban food and nutrition security while also exposing the systemic fragilities these actors face. The study also contributes methodologically by applying a robust regression approach to account for heteroskedasticity and outliers in a small sample, an important consideration for future research in informal economies where data irregularities are common. The findings reinforce that while indigenous foods hold immense nutritional and cultural value, their market viability is undermined by infrastructural deficits, volatile customer demand, and frequent exposure to overlapping shocks such as flooding, looting, and pandemics. Key policy implications include the need to invest in decentralized storage infrastructure, subsidized market rentals, and vendor cooperatives to pool logistics costs. Such interventions could reduce operational fragility and enhance the resilience of informal food systems.

These findings have important implications for post-disaster policy planning and vendor resilience support in urban informal markets. Specifically, the eThekwini Municipality could draw on these insights to strengthen its vendor support mechanisms by:

- Establishing targeted emergency relief funds for vendors affected by climatic and market shocks.
- Investing in mobile or shared cold storage infrastructure within the Warwick Early Morning Market to reduce postharvest losses.
- Creating and maintaining vendor disaster registries to enable timely and efficient aid distribution; and
- Integrating informal vendors into the city’s disaster preparedness frameworks and food security governance platforms.

These interventions would not only cushion vendors against future income shocks but also enhance their adaptive capacity contributing to broader development objectives such as SDG 13 (Climate Action) and SDG 2 (Zero Hunger).

### 6.1 Study limitations

This study offers important insights into the income vulnerability of indigenous crop vendors in Durban’s informal food economy; however, several limitations must be acknowledged.

First, the sample size (n = 34) is relatively small and geographically limited to a single urban market in Warwick Early Morning Market. While the sample accurately reflects the population of formally registered indigenous crop vendors in that location, it limits the generalizability of findings to other urban or national settings. The analysis should thus be interpreted as context-specific rather than broadly representative. Second, the study employs a cross-sectional design, which restricts our ability to establish causal relationships or observe temporal patterns in vendor resilience and recovery. A longitudinal or panel-based approach would be better suited to examine adaptation trajectories over time. Third, the study relied on self-reported income loss, which may be subject to recall bias especially given that participants were reflecting on multiple overlapping shocks (COVID-19, flooding, unrest). While efforts were made to minimize bias through careful questionnaire design and field training, some inconsistencies in recall or attribution are possible. Finally, while robust regression methods were used to address small sample heteroskedasticity, the study did not include a composite index of vulnerability or detailed analysis of adaptive strategies. Future research could benefit from a mixed-methods approach combining quantitative indicators with qualitative insights to better understand how vendors perceive and respond to systemic shocks.

## Data Availability

The datasets presented in this article are not readily available because this is part of a collaboration with the municipality, and the data contains sensitive personal information of street vendors. Requests to access the datasets should be directed to Xolile Mkhize, xmkhize@mut.ac.za.

## Appendix

**Table:**

Glossary of acronyms and indigenous crop names	
Term/Acronym	Full name/description
SDG	Sustainable Development Goals
IFC	Indigenous Food Crops
COVID-19	Coronavirus Disease 2019
KZN	KwaZulu-Natal (Province in South Africa)
EMA	Early Morning Market
CHO	Carbohydrate
Ca	Calcium
Fe	Iron
IU	International Units (for vitamins).

**Table:**

Indigenous crop names		
Local name (isiZulu)	Scientific name	Common name
Ubhatata	*Ipomoea batatas*	Sweetpotato
Amadumbe	*Colocasia esculenta*	Taro
Imbuya	*Amaranthus* spp.	Pigweed
Imfino yezintanga	*Cucurbita maxima* (leaves)	Pumpkin leaves
Intshungu	*Momordica foetida*	Bitter Cucumber/African Vine
Amantongomane	*Arachis hypogaea*	Groundnuts/Peanuts
Umdumbulu	*Manihot esculenta*	Cassava
